# Prophylactic Mesh Placement for the Prevention of Incisional Hernia in High-Risk Patients After Abdominal Surgery: A Systematic Review and Meta-Analysis

**DOI:** 10.7759/cureus.10491

**Published:** 2020-09-16

**Authors:** Jawad Ahmed, Nimra Hasnain, Iayla Fatima, Farheen Malik, Muhammad A Chaudhary, Junaid Ahmad, Mehreen Malik, Laraib Malik, Muhammad Osama, Mirza Zain Baig, Faisal Khosa, Faiz Bhora

**Affiliations:** 1 Internal Medicine, Dow University of Health Sciences, Karachi, PAK; 2 General Surgery, St. Luke’s General Hospital, Killenny, IRL; 3 Center for Surgery and Public Health, Harvard Medical School/Harvard T. H. Chan School of Public Health, Boston, USA; 4 Family Medicine, WellSpan Good Samaritan Hospital, Lebanon, USA; 5 Liaquat Medical College, Liaquat University of Medical and Health Sciences, Jamshoro, PAK; 6 Anesthesiology, Aga Khan University, Karachi, PAK; 7 Pediatrics, Abbasi Shaheed Hospital, Karachi, PAK; 8 General Surgery, Dow University of Health Sciences, Karachi, PAK; 9 Surgical Oncology, Health Quest System, New York, USA; 10 Radiology, Vancouver General Hospital, Vancouver, CAN; 11 Thoracic Surgery, Health Quest System, New York, USA

**Keywords:** incisional hernia, mesh placement, suture, chronic wound pain, seroma, prophylactic mesh use, high-risk, abdominal surgery, laparotomy, laparoscopic surgery

## Abstract

Background and objectives

In high-risk populations, the efficacy of mesh placement in incisional hernia (IH) prevention after elective abdominal surgeries has been supported by many published studies. This meta-analysis aimed at providing comprehensive and updated clinical implications of prophylactic mesh placement (PMP) for the prevention of IH as compared to primary suture closure (PSC).

Materials and methods

PubMed, Science Direct, Cochrane, and Google Scholar were systematically searched until March 3, 2020, for studies comparing the efficacy of PMP to PSC in abdominal surgeries. The main outcome of interest was the incidence of IH at different follow-up durations. All statistical analyses were carried out using Review Manager version 5.3 (The Nordic Cochrane Centre, The Cochrane Collaboration, 2014) and Stata 11.0 (Stata Corporation LP, College Station, TX). The data were pooled using the random-effects model, and odds ratio (OR) and weighted mean differences (WMD) were calculated with the corresponding 95% confidence interval (CI).

Results

A total of 3,330 were identified initially and after duplicate removal and exclusion based on title and abstract, 26 studies comprising 3,000 patients, were included. The incidence of IH was significantly reduced for PMP at follow-up periods of one year (OR= 0.16 [0.05, 0.51]; p=0.002; I^2^=77%), two years (OR= 0.23 [0.12, 0.45]; p<0.0001; I^2^=68%), three years (OR= 0.30 [0.16, 0.59]; p=0.0004; I^2^= 52%), and five years (OR=0.15 [0.03, 0.85]; p=0.03; I^2^=87%). However, PMP was associated with an increased risk of seroma (OR=1.67 [1.10, 2.55]; p= 0.02; I^2^=19%) and chronic wound pain (OR=1.71 [1.03, 2.83]; p= 0.04; I^2^= 0%). No significant difference between the PMP and PSC groups was noted for postoperative hematoma (OR= 1.04 [0.43, 2.50]; p=0.92; I^2^=0%), surgical site infection (OR=1.09 [0.78, 1.52]; p= 0.62; I^2^=12%), wound dehiscence (OR=0.69 [0.30, 1.62]; p=0.40; I^2^= 0%), gastrointestinal complications (OR= 1.40 [0.76, 2.58]; p=0.28; I^2^= 0%), length of hospital stay (WMD= -0.49 [-1.45, 0.48]; p=0.32; I^2^=0%), and operating time (WMD=9.18 [-7.17, 25.54]; p= 0.27; I^2^=80%).

Conclusions

PMP has been effective in reducing the rate of IH in the high-risk population at all time intervals, but it is associated with an increased risk of seroma and chronic wound pain. The benefits of mesh largely outweigh the risk, and it is linked with positive outcomes in high-risk patients.

## Introduction

Any procedure that requires access to the abdominal wall carries with itself the precarious complication of incisional hernia (IH). This is especially common in patients undergoing open bariatric surgery and abdominal aneurysm repair. The incidence of IH is approximately 11%-20% in patients post laparotomy, but it can be as high as 39.9% in high-risk populations such as obesity, prior abdominal operation, abdominal aortic aneurysm, or patients suffering from neoplastic diseases [1-4]. Annually, 150,000 patients are operated for IH in the United States alone, with one-third repairs occurring within nine years. IH not only creates a financial burden but also leads to poor health-related quality of life (QoL) in patients. It is also associated with poor body image and a lower sense of self-worth [2,5-6].

Mesh placement has been found effective in reducing occurrences of umbilical hernia, inguinal hernia, and parastomal hernia. Previous systematic reviews have also yielded supportive findings regarding the efficacy of prophylactic mesh placement (PMP) in preventing IH [4,7-8]. However, they did not evaluate the time-based effectiveness of PMP as compared to primary suture closure (PSC) and did not study the differences among various population subgroups, as they were limited by small sample size. Furthermore, the literature remains inconclusive on whether the mesh is efficacious in reducing chronic wound pain [8].

Due to the lack of sufficient quality evidence, there is a need for further high-quality studies to support the use of mesh for IH prevention in high-risk patients [8]. Several new studies have been published since the last meta-analysis, and therefore, we sought to conduct an updated meta-analysis of all studies to date. The larger sample size enabled us to provide a holistic, well-powered assessment of the efficacy of a prophylactic mesh in preventing IH. One of the reasons why the efficacy of a prophylactic mesh has remained unclear is maybe because of varying effectiveness in different patient subgroups. Hence, we also aimed to conduct a range of subgroup analyses to identify specific patient populations in which a prophylactic mesh might be beneficial. Additionally, we sought to evaluate seldom-evaluated aspects of mesh placement, including hematoma, seroma, chronic wound pain, surgical site infections, gastrointestinal complications, operating time, and length of hospital stay.

## Materials and methods

The current study has been carried out in accordance with the Preferred Reporting Items for Systemic Reviews and Meta-analysis (PRISMA) guidelines. Two independent reviewers carried out the literature search, quality assessment, data extraction, and statistical analyses. In case of any conflict, a third reviewer was consulted.

Search strategy

Online databases, including PubMed, Science Direct, and CENTRAL Register of Controlled Trials (Cochrane), were systematically searched from the inception of databases till March 3, 2020, without time or language restrictions. Google Scholar was also searched for gray literature. References of relevant reviews were also manually searched for additional studies. The search strategy for each database is given in Table 1.

**Table 1:** Search strategy for online databases

Online databases	Search strategy
PubMed	((((((mesh[tiab] OR prosthe*[tiab] OR implant*[tiab]))) AND ((prophyla*[tiab] OR prevent*[tiab]))) AND herni*[tiab]) AND ((incision*[tiab] OR postoperat*[tiab] OR laparotomy[tiab] OR laparoscopy*[tiab] OR surger*[tiab] OR surgic*[tiab] OR operation*[tiab] OR operative*[tiab] OR ventral*[tiab] OR transverse*[tiab] OR abdom*[tiab])))
Google Scholar	incisional hernia AND prophylactic AND mesh repair OR mesh placement AND midline laparotomy OR laparoscopic surgery AND suture closure
Cochrane	prophylactic AND mesh AND incisional hernia
Science Direct	incisional hernia AND prophylactic AND mesh repair OR mesh placement AND midline laparotomy AND laparoscopic surgery AND suture closure

Study selection

All the studies were imported into EndNote Reference Library version X4 (Clarivate Analytics, Thomson Reuters Corporation, Philadelphia, Pennsylvania), and duplicates were screened and removed. Randomized controlled trials (RCTs) and observational studies encompassing all patients >18 years undergoing an elective laparotomy or laparoscopic procedure and ≥ 1 risk factor for incisional hernia (prior abdominal operation, neoplastic disease, history of abdominal aortic aneurysm, ≥45 years of age, body mass index (BMI) ≥25 kg/m^2^, smoking, and chronic obstructive pulmonary disease history) were included.

Exclusion criteria included primary or prior surgery for hernia or existing abdominal mesh, emergency cases, life expectancy <24 months, and pre-existing pregnancy. Studies without a control group and all procedures done for mesh placement in stoma sites were excluded as well. Only elective cases were considered and studies reporting emergency surgeries were excluded.

Data extraction and outcomes

Information regarding study characteristics, demographics, and reported outcomes were extracted. Four different mesh locations were considered: (1) Onlay position (above the anterior rectus sheath or below the abdominal fascia); (2) Retrorectus, also known as the sublay position (between the rectus abdominis muscle and posterior rectus sheath); (3) Preperitoneal (between the posterior rectus sheath and parietal peritoneum), and (4) Intraperitoneal (in the abdominal cavity).

The main outcome of interest was the incidence of IH at different follow-up durations. Other outcomes included seroma, chronic wound pain, hematoma, wound dehiscence, surgical site infection, respiratory and gastrointestinal complications, hospital stay, and operating time. The incidence of IH was confirmed by clinical examination or imaging modalities, such as ultrasonography or computed tomography (CT) scan, and no difference was made between IH diagnosed clinically or through imaging modalities. Gastrointestinal complications included ascites, bowel obstruction, bowel perforation, intra-abdominal abscess, and paralytic ileus. We accepted the primary study investigator’s definition for seroma and all remaining outcomes.

The number of patients that presented during follow-up was considered as the denominator instead of randomization numbers for meta-analyses of outcomes. Studies were classified in each follow-up group based on follow-up time. Where specific follow-up was not mentioned, mean or median follow-up was used to classify the study. Study characteristics and early complications were extracted from earlier publications of a trial if publication of the latest follow-up data lacked them. The incidence of IH was recorded from each follow-up duration. Studies that did not provide means and standard deviations (SD) for hospital stay duration and operation time were not included in the respective analysis.

Statistical analysis

Review Manager v.5.3 (The Nordic Cochrane Centre, The Cochrane Collaboration, 2014) and Stata 11.0 (Stata Corporation LP, College Station, TX) were used for all statistical analyses.

Patients’ data were divided into two groups - PSC or PMP - according to the procedure. Weighted mean differences (WMD) and Mantel-Haenszel (MH) odds ratios (OR) were calculated with a 95% confidence interval (CI) and pooled using a random-effects model. Subgroup analyses were performed by stratifying studies according to study design (RCT and observational), mesh location (onlay, retrorectus, preperitoneal, and intraperitoneal), BMI (<40 and >40), and study population (bariatric, neoplastic, vascular, and mixed). The chi-squared test (p-interaction) was used to assess subgroup differences.

Statistical heterogeneity was quantified across studies using Higgin's I^2^ statistics, and a value of 25%-50% was considered mild, 50%-75% as moderate, and >75% as severe. The leave one out analysis was performed to determine whether any single study had a disproportionate effect on the pooled results.

Quality assessment

The quality assessment of studies was done using the Cochrane Collaboration risk-of-bias tool and the Newcastle-Ottawa scale for RCTs and observational studies, respectively. Publication bias was assessed using a funnel plot and Egger’s regression test. A p-value of ≤ 0.05 was considered significant in all cases.

## Results

**Figure 1:**
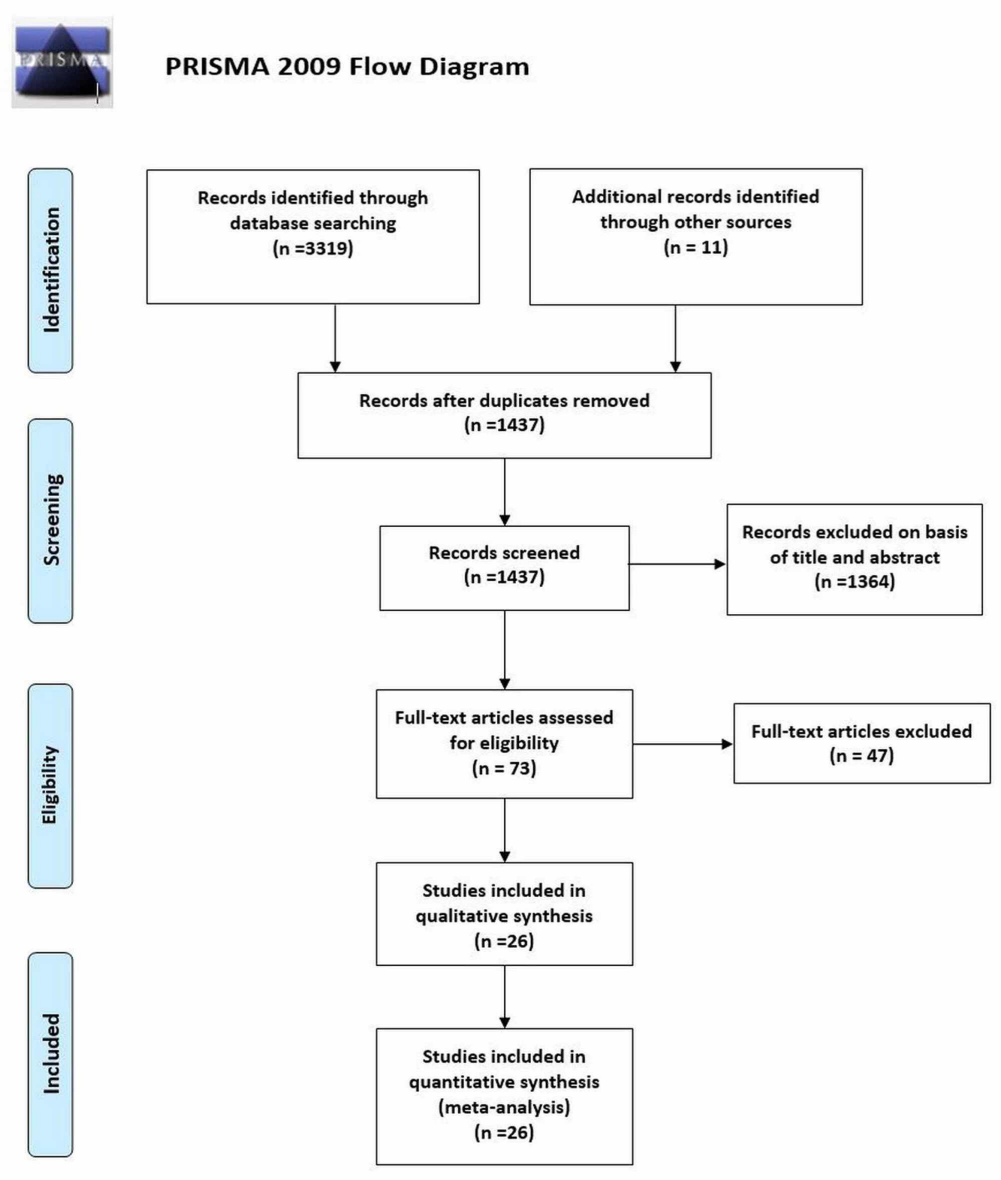
PRISMA flow diagram for literature search PRISMA, Preferred Reporting Items for Systemic Reviews and Meta-Analysis

A total of 3,330 records were identified in the initial search, 3,319 from electronic databases and 11 through references of relevant studies (other sources). After removing duplicates and excluding articles based on title and abstract screening, the full texts of 73 articles were reviewed for eligibility. A total of 26 articles met the inclusion criteria [1-3,5,9-29]. Figure 1 shows the PRISMA flowchart summarizing the literature search.

The follow-up time in the included studies ranged from six to 60 months. Out of the 26 studies, 17 were RCTs and nine were observational (seven prospective cohorts and two retrospective studies). The included studies enrolled a total of 3,349 participants, from which 3,000 were analyzed (1,397 receiving PMP and 1,603 receiving PSC). The rest were either lost to follow-up or excluded during surgery. Study characteristics and demographics are given in Table 2.

**Table 2:** Baseline characteristics and demographics of included studies PSC, primary suture closure; PMP, prophylactic mesh placement; IH, incisional hernia; RCT, randomized controlled trial; BMI, body mass index * These studies have longer duration results published separately [26-29]; thus 22 studies are shown in this table. In studies where multiple follow-up intervals are reported, only the incidence of IH at the latest follow-up is shown.

Study; Year; Location; Study design	Study population	Total no. of patients; Males (%); Age in years (SD); BMI in kg/m^2^		Type of Incision & surgery	Cohort	N	No. of IH (%)	Incidence reporting
		MESH	NO MESH					
Pans, 1998 [9]; Belgium; RCT	Bariatric	144; 41 (28.4); 36.6 (0.9); 43.8 (0.5)	144; 30 (20.8); 36.4 (0.9); 43.7(0.6)	Midline incision; Open bariatric surgery	PSC	144	41 (28.5)	0-67 months (mean follow-up was 29.8)
					PMP -intraperitoneal	144	33 (22.9)	
Strzeczyk, 2002 [10]; Poland; Prospective	Bariatric	12 (mesh) vs 48 (non-mesh); 37 (61.7); 37.3 (11.2); 45.1 (7.2).		Midline incision; Open Roux-en-Y gastric bypass surgery	PSC	48	9 (18.8)	12 months
					PMP - onlay	12	0 (0.0)	
Peña, 2003 [11]; Spain; RCT	Neoplastic and high risk	50 (mesh) and 50 (non-mesh); 67 (67); 64.3 (42-83).		Medial and paramedial incision; Laparotomy	PSC	44	5 (11.4)	36 months
					PMP - onlay	44	0 (0.00)	
Strzelczyk, 2006 [12]; Poland; RCT	Bariatric	37; 24 (66.7); 39.4(12.3); 46.2 (7.1)	40; 23 (60.5); 38.9(11.8); 46.8(7.6)	Midline incision; Open Roux-en-Y gastric bypass surgery	PSC	38	8 (21.1)	6-38 months (mean 28 months)
					PMP -retrorectus	36	0 (0.0)	
El- Khadrawy, 2009 [1]; Egypt; RCT	Bariatric	20; 8 (40); 47.86 (13.82); 9 (45%) obese	20; 10 (50); 47.61 (14.11); 8 (40) obese	Midline incisions; Abdominal operation	PSC	20	3 (15)	36 months
					PMP - preperitoneal	20	1 (5)	
Bevis, 2010 [13]; UK; RCT	Abdominal aortic aneurysm	40; 34 (85); 74 (59-84)	45: 43 (95.5); 72 (59-89)	Midline incision; Open abdominal aortic aneurysm repair	PSC	43	16 (37.2)	36 months (mean follow-up 26)
					PMP - retrorectus, preperitoneal	37	5 (13.5)	
Llaguna, 2011 [14]; USA; Prospective	Bariatric	59; 13 (29.55); 43.73 (11.81); 52.58 (10.59	75; 10 (16.13); 39.39 (11.08); 50.38 (9.31)	Midline incision; Open Roux-en-Y gastric bypass surgery	PSC	62	11 (17.7)	24 months
					PMP -preperitoneal	44	1 (2.3)	
Curro, 2012* [15]; Italy; Prospective	Bariatric	45; 7 (15.5); 38 (27-64); 45 (40-60)	50; 9 (18); 39 (23-66); 46(40-65)	Midline incision; Open biliopancreatic diversion	PSC	50	15 (30)	12 and 24 months
					PMP - retrorectus	45	2 (4.4)	
Abo-Ryia, 2013 [16]; Egypt; RCT	Bariatric	32; 6 (18.7); 38.5 (10.8); 52.2 (9.1)	32; 7 (21.8); 36.9 (11.3); 51.4 (10.5)	Midline incision; Open bariatric surgery	PSC	32	9 (28.1)	6, 12,18 and 24 months
					PMP - preperitoneal	32	1 (3.1)	
Armañanzas, 2014 [17]; Spain; RCT	Symptomatic cholelithiasis and high risk	53; 11(24.4); 60.3 (16.2); 30.5 (6.1)	53; 9 (19.1); 61.9 (15.3); 30.6 (5.3)	Laparoscopic cholecystectomy	PSC	47	15 (31.9)	24 hours and 12 months
					PMP -intraperitoneal	45	2 (4.4)	
Sarr, 2014 [18]; USA; RCT	Bariatric	199; 39 (21); 44.6(10.6); 48.2 98.2)	203; 39 (20); 45.1 (12.1); 48.2(7.7)	Midline incision; Open Roux-en-Y gastric bypass surgery	PSC	195	38 (19.5)	6, 12 and 24 months
					PMP - preperitoneal	185	32 (17.3)	
Bali, 2015 [19]; Greece; RCT	Abdominal aortic aneurysm	20; 18 (90); 75; 25.4	20; 18 (90); 75; 24.4	Midline incision; Open abdominal aortic aneurysm repair	PSC	20	6 (30)	36 months
					PMP - onlay	20	0 (0.0)	
Muysoms, 2016 [20]; Belgium; RCT	Abdominal aortic aneurysm	56; 54 (96); 72 (7.4); 25 (3.6)	58; 51 (88); 72 (8.5); 26 (3.7)	Midline incision; Open abdominal aortic aneurysm repair	PSC	58	16 (27.6)	12 and 24 months
					PMP - retrorectus	56	0 (0.0)	
Blázquez, 2016 [21]; Spain; Prospective	Neoplastic	58; 35 (60.3); 62.59 (11); 27.33 (5.68)	57; 35 (61.4); 61.96 (12); 28.35 (5.40)	Bilateral subcostal incisions; Abdominal operations	PSC	57	10 (17.54)	24 months
					PMP - onlay	58	1 (1.72)	
Jairam, 2017* [5]; Netherlands, Germany, and Austria; RCT	Abdominal Aortic Aneurysm	PSC- 107; 68 (64); 65.2 (10.5); 29.8 (4.4)		Midline incision; Open abdominal aortic aneurysm repair	PSC	107	33 (30)	24 months
		Onlay mesh- 188; (62); 64.2 (12.3); 30.8 (5.9)			PMP- onlay	188	25 (13)	
		Retrorectus mesh – 185; (58); 64.4 (10.4); 30.8 (5.2)			PMP - retrorectus	185	34 (18)	
Hoyuela, 2017 [22]; Spain; Prospective	Neoplastic	15; 10 (66.7); 76.4 (11); 27.8 (2)	37; 23 (62.2); 71 (11); 28.9 (2)	Laparoscopic colon resection	PSC	37	4 (10.8)	18 months
					PMP - onlay	15	0 (0.0)	
Kohler, 2018 [23]; Switzerland; RCT	Neoplastic or high risk	83; 46 (66.7); 67 (58-72); 27.6 (4.6)	86; 56 (69.1); 65 (56.5-70); 26.7 (4.8)	Midline or transverse incision; Open abdominal surgery	PSC	81	15 (18.5)	36 months
					PMP-intraperitoneal	69	5 (7.2)	
Argudo, 2018 [24]; Spain; Prospective	Neoplastic	226; 138 (61); 77 (11)		Midline incision; Open abdominal surgery	PSC	114	36 (31.6)	12- 60 months (mean 32 months)
					PMP - onlay	112	9 (8)	
Pereira, 2018 [25]; Spain; Retrospective	Neoplastic	Midline Incision without mesh- 61; 40 (65.6); 69.3 (12.5); 26.6 (4.4)		Midline or transverse incision; Laparoscopic colon and rectal resection	PSC - midline incision (no mesh)	61	20 (32.8)	Up to 20 months (median 13 months)
		Transverse incision- 87; 50 (57.5); 68.8 (11.8); 26.3 (4.2)			PSC - transverse incision	87	16 (18.4)	
		Midline incision with mesh-34; 17 (50); 72.4 (10.9); 30.2 (5.6)			PMP - onlay	34	3 (8.8)	
Rhemtulla, 2018 [6]; USA; Retrospective	High risk	18; 8 (44.4); 54.3; 29.5	75; 35 (46.7); 58.2; 29.5	Midline incision; Abdominal laparotomy	PSC	75	4 (5.3)	6 months
					PMP - onlay	18	0 (0.0)	
Glauser, 2019* [2]; Switzerland; RCT	High risk	131; 60 (45.8); 64.1 (61.9- 66.4); 25.8 (25.0-26.7)	136; 56 (41.2); 65.1 (63.1- 67.1); 26.6 (25.8-27.4)	Midline incision; Abdominal surgery/laparotomy	PSC	88	46 (52.3)	24 and 60 months
					PMP - intraperitoneal	95	26 (27.4)	
Caro‑Tarrago, 2019* [3]; Spain; RCT	Neoplastic	80; 44 (55); 64.32 (14.27); >30 (26.3)	80; 46 (57.5); 67.32 (11.11); >30 (30.1)	Midline incision; Abdominal surgery/laparotomy	PSC	80	37 (46.8)	12 and 60 months
					PMP - onlay	80	4 (5.1)	

Mesh, suture, and surgery details

In all except four studies, patients underwent midline laparotomy. In three studies, laparoscopic surgery was done, and in one study, patients underwent bilateral subcostal incisions [17,21-22,25]. A variety of meshes were used in the studies, with polypropylene (PP) being the most common one (n=11 studies). Three studies used different biologic meshes, i.e., Alloderm, Surgisis Gold, and Bovine pericardium [14,18-19]. One study used an unspecified biosynthetic mesh [6]. Other meshes used included, but were not limited to, polyglactin, propylene polyglycolic acid, and polypropylene-polyvinylidene fluoride. Meshes were placed in four different locations. Two studies planted mesh in two separate locations [5,13]. Nine studies placed the mesh in the onlay position. The most commonly used technique in studies for aponeurosis closure was continuous. The diagnostic modalities for IH, mesh, and suture details for included studies are summarized in the Appendices section.

Quality assessment and publication bias

**Figure 2:**
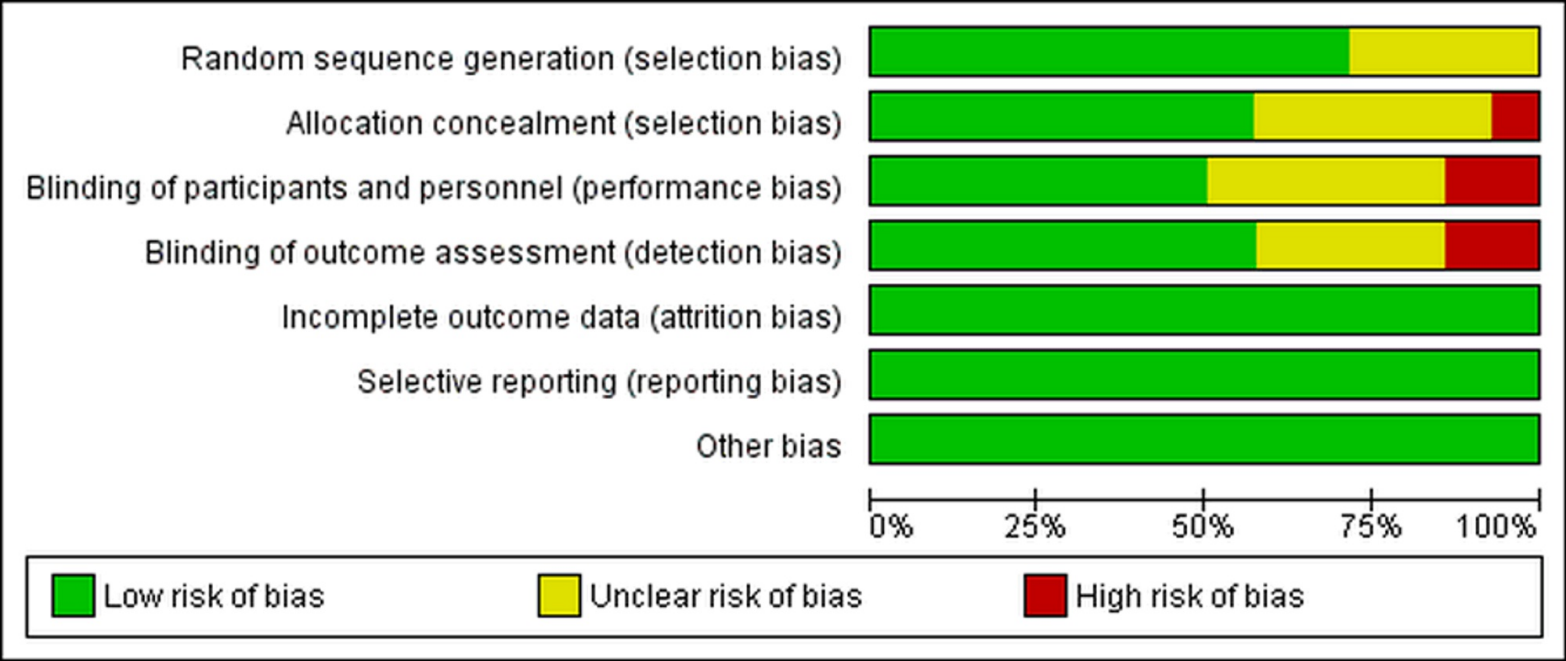
Assessment of publication bias in randomized controlled trials using the Cochrane Collaboration risk-of-bias tool

**Figure 3:**
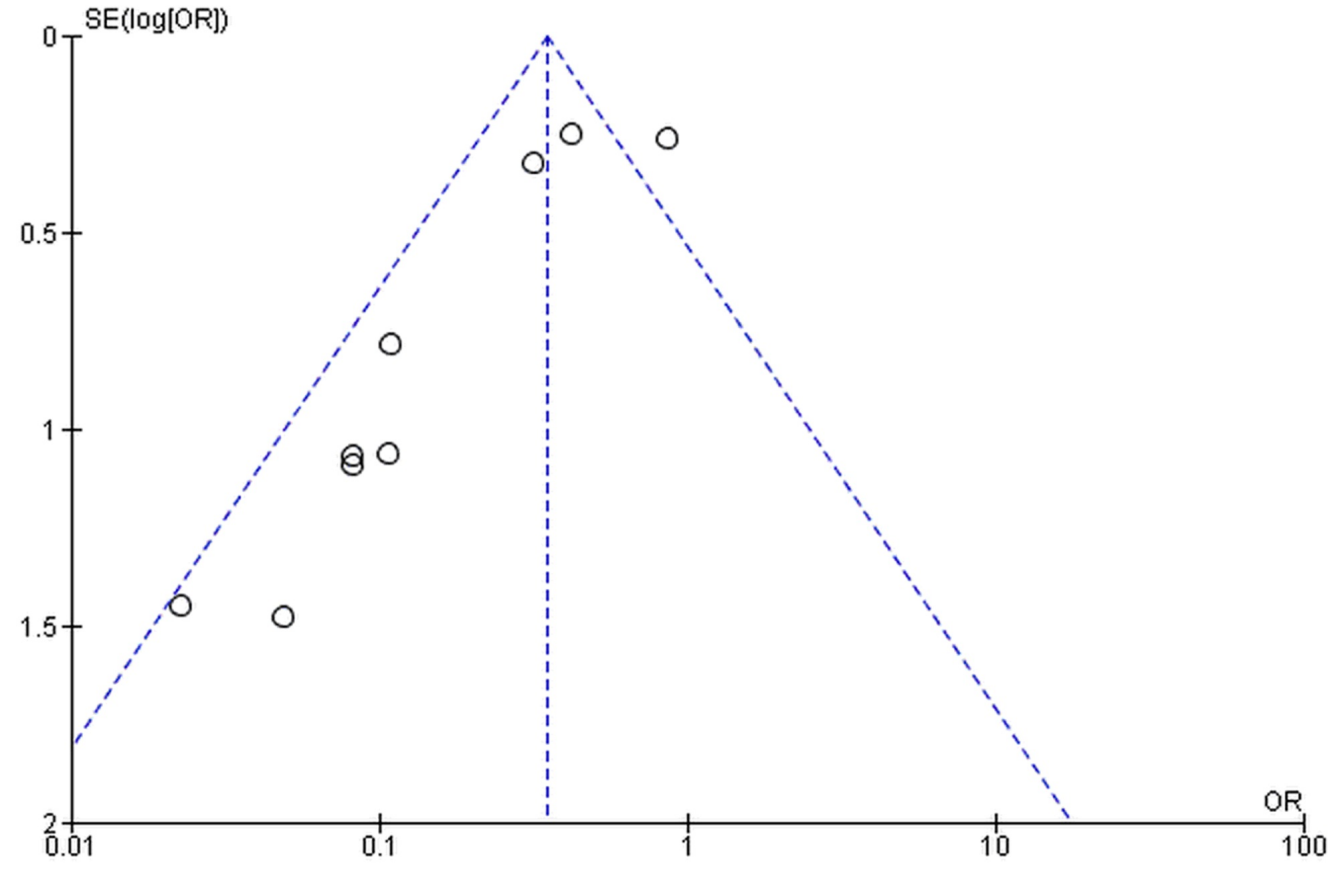
Funnel plot for publication bias Funnel plot is based on the two-year incisional hernia outcome follow-up. SE, standard error; OR, odds ratio

The majority of the RCTs and observational studies were of robust methodological quality. Half of the RCTs either had a high or unclear risk of bias in the blinding of participants and personnel (Figure 2). Details of bias assessment in observational studies are present in Table 3. The funnel plot showed significant publication bias (Figure 3), and it was confirmed by Egger’s regression test (p=0.031).

**Table 3:** Quality assessment of observational studies included in the meta-analysis using the New-Castle Ottawa scale

Author, year	Selection				Comparability	Outcome			Total score
	Representativeness of the exposed cohort	Selection of non-exposed cohort	Ascertainment of exposure	Demonstration that outcome was not present at the beginning	Comparability of groups	Assessment of outcome	Was follow up long enough for outcomes to occur?	Adequacy of follow up of cohorts	
	High-risk population	High-risk population	Surgery record on databases	Surgery record on databases	-	Blinded and independent	≥6 months	≥90%	
Curro, 2012 [15]	1	1	1	1	1	0	1	1	7
Llaguna, 2011 [14]	1	1	1	1	1	0	1	0	6
Strzeczyk, 2002 [10]	1	1	1	1	1	0	1	1	7
Argudo, 2018 [24]	1	1	1	1	1	1	1	1	8
Pereira, 2018 [25]	1	1	1	1	1	1	1	1	8
Blázquez-Hernando, 2016 [21]	1	1	1	1	1	1	1	1	8
Hoyuela, 2017 [22]	1	1	1	1	1	1	1	1	8
Rhemtulla, 2018 [6]	1	1	1	1	1	1	1	1	8

Results of meta-analyses

**Figure 4:**
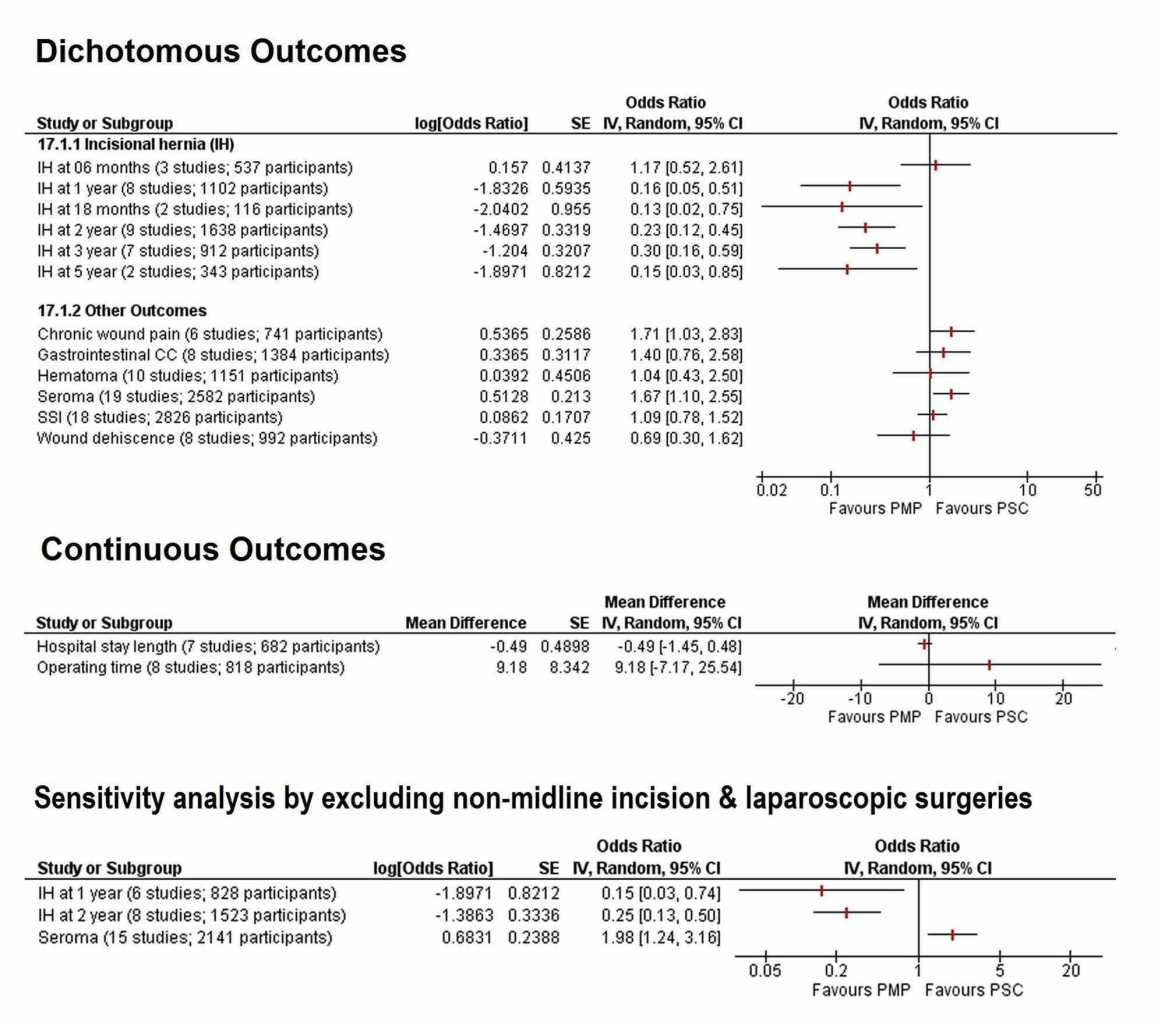
Forest plot summarizing the results of all the meta-analyses CC, complication; CI, confidence interval; IV, inverse variance; M-H, Mantel-Haenszel; PMP, prophylactic mesh placement; PSC, primary suture closure; SSI, surgical site infection

The summarized results of all outcomes discussed below are given in Figure 4. Individual outcomes with their forest plots are given under their respective subheadings. A table summarizing the effects of the leave one out analysis for each outcome is given in the appendices section.

Incidence of IH at six months

Three studies (PMP - 235 patients, 13 events; PSC - 302 patients, 15 events) mentioned IH occurrence at the six-months follow-up. No significant difference was found between the PMP and PSC groups (OR=1.17 [0.52, 2.61]; p=0.71; I^2^=0%). The leave one out analysis did not reveal any single study, which had a disproportionate effect on the results.

**Figure 5:**
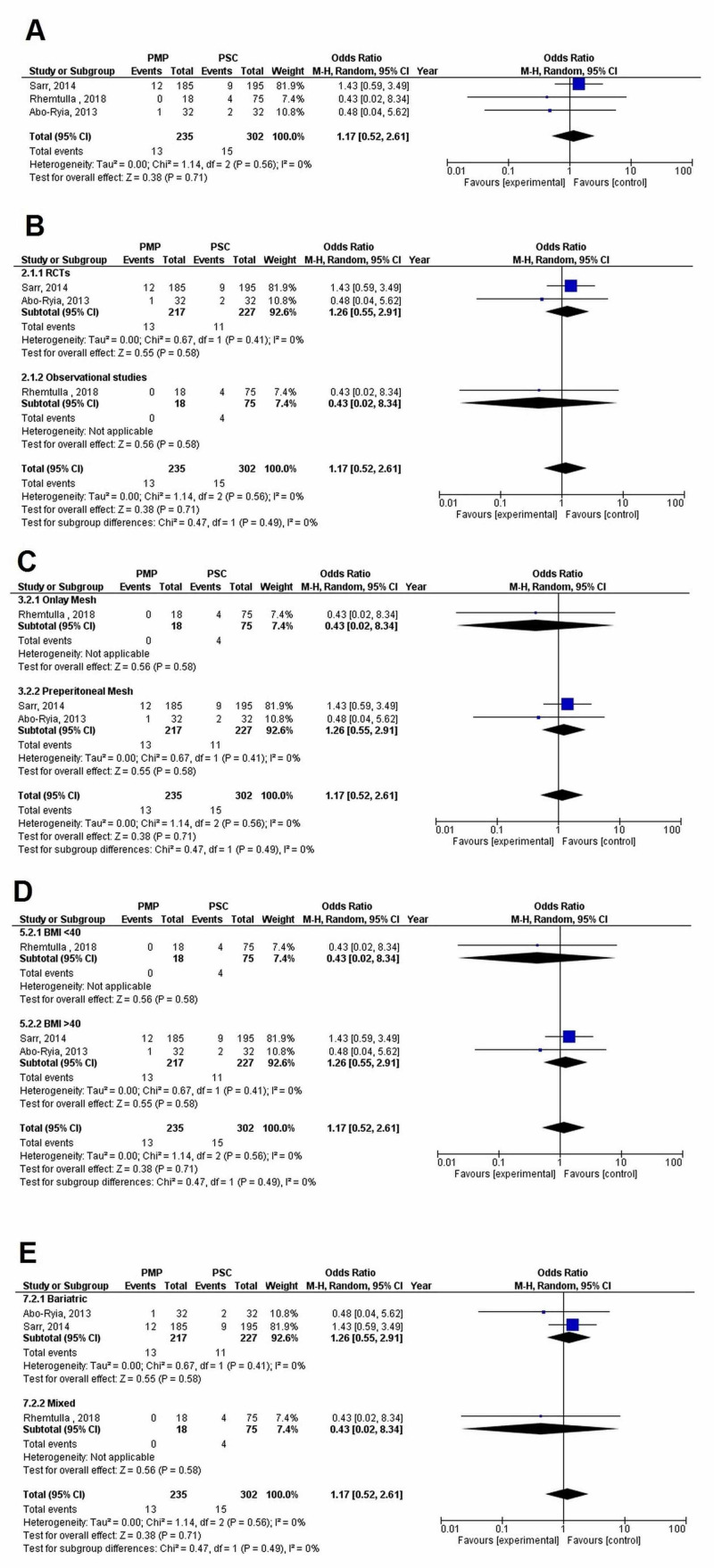
Incisional hernia at six months (A) Overall analysis; (B) Subgroups by study design; (C) Subgroups by mesh location; (D) Subgroups by BMI; and (E) Subgroups by population PMP, prophylactic mesh placement; PSC, primary suture closure; CI, confidence interval; M-H, Mantel-Haenszel Studies used in the analyses include [6,16,18].

There was no significant difference in the incidence of IH between subgroups when data was stratified according to (1) Study design (p-interaction=0.49; I^2^=0%), (2) Mesh location (p-interaction=0.49; I^2^=0%), (3) BMI (p-interaction=0.49; I^2^=0%), or (4) Study population (p-interaction=0.49; I^2^=0%) as shown in Figure 5.

Incidence of IH at one year

A total of eight studies (PMP - 469 patients, 37 events; PSC - 633 patients, 141 events) reported IH incidence at the one-year follow-up. PMP significantly reduced the incidence of IH when compared to PSC (OR=0.16 [0.05, 0.51]; p=0.002; I^2^=77%). Sensitivity analysis did not reveal any disproportionate effects. Notably, however, heterogeneity (I^2^) dropped to 0% on removing the Sarr, 2014, study.

No significant difference was found between subgroups upon stratifying data according to (1) Study design (p-interaction=0.70; I^2^=0%), (2) Mesh location (p-interaction=0.28; I^2^=21.1%), (3) BMI (p-interaction=0.11; I^2^=60.2%), and (4) Study population (p-interaction=0.58; I^2^=0%).

**Figure 6:**
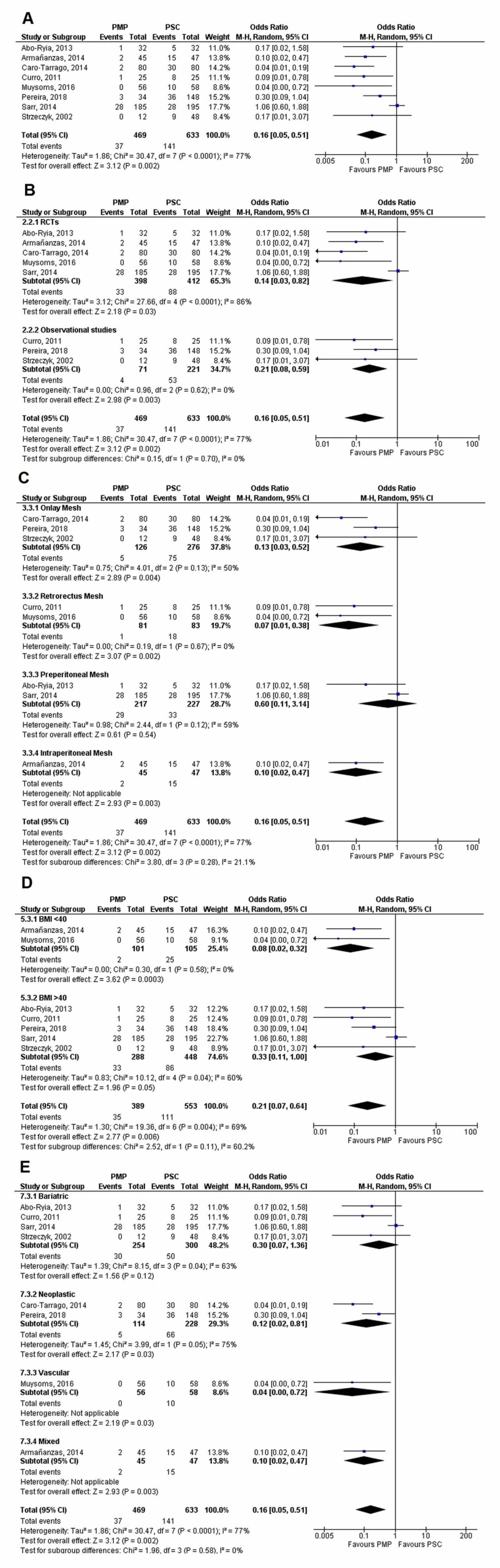
Incisional hernia at one year (A) Overall analysis; (B) Subgroups by study design; (C) Subgroups by mesh location; (D) Subgroups by BMI; and (E) Subgroups by population PMP, prophylactic mesh placement; PSC, primary suture closure; CI, confidence interval; M-H, Mantel-Haenszel Studies used in the analyses include [10,15-18,20,25,29].

It was noted that upon subgroup analysis by the study population, PMP significantly reduced the IH risk in all study populations except bariatric (OR=0.30 [0.07, 1.36]; p=0.12; I^2^=63%). All forest plots are given in Figure 6.

Table 4 highlights the different subgroups analysis carried out for IH at the six-month and one-year follow-ups.

**Table 4:** Results of subgroup analyses for IH at the six-month and one-year follow-ups All outcomes are stratified according to study design (RCTs or observational), mesh location (onlay, retrorectus, preperitoneal, and intraperitoneal), mean BMI (<40 and >40), and study population (bariatric, neoplastic, vascular, and mixed). The value of I^2^ shows the heterogeneity among subgroups. P_subgroup_ represents p-values between subgroups. IH, incisional hernia; OR, odds ratio; No Sig. Diff., no significant difference; Sig. Diff., Significant difference.

Subgroups	IH at 6-months follow-up					IH at 1-year follow-up				
	N studies	I^2^ (%)	OR [95% CI]	P_subgroup_	Comments	N studies	I^2^ (%)	OR [95% CI]	P_subgroup_	Comments
Study design										
RCT	2	0	1.26 [0.55, 2.91]	0.49	No Sig. Diff.	5	86	0.14 [0.03, 0.82]	0.70	No Sig. Diff.
Observational	1	-	0.43 [0.02, 8.34]			3	0	0.21 [0.08, 0.59]		
Mesh location										
Onlay	1	-	0.43 [0.02, 8.34]	0.49	No Sig. Diff.	3	50	0.13 [0.03, 0.52]	0.28	No Sig. Diff.
Retrorectus	-	-	-			2	0	0.07 [0.01, 0.38]		
Preperitoneal	2	0	1.26 [0.55, 2.91]			2	59	0.60 [0.11, 3.14]		
Intraperitoneal	-	-	-			1	-	0.10 [0.02, 0.47]		
Mean BMI										
<40	1	-	0.43 [0.02, 8.34]	0.49	No Sig. Diff.	2	0	0.08 [0.02, 0.32]	0.11	No Sig. Diff.
>40	2	0	1.26 [0.55, 2.91]			5	60	0.33 [0.11, 1.00]		
Study population										
Bariatric	2	0	1.26 [0.55, 2.91]	0.49	No Sig. Diff.	4	63	0.30 [0.07, 1.36]	0.58	No Sig. Diff.
Neoplastic	-	-	-			2	75	0.12 [0.02, 0.81]		
Vascular	-	-	-			1	-	0.04 [0.00, 0.72]		
Mixed	1	-	0.43 [0.02, 8.34]			1	-	0.10 [0.02, 0.47]		

Incidence of IH at 18 months

**Figure 7:**
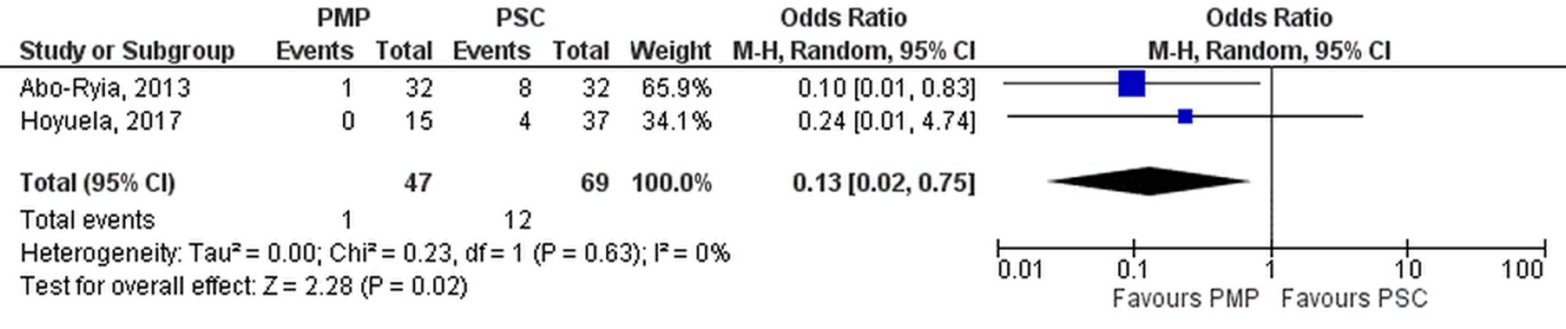
Incisional hernia at 18 months PMP, prophylactic mesh placement; PSC, primary suture closure; CI, confidence interval; M-H, Mantel-Haenszel Studies used in the analysis include [16,22].

Two studies (PMP- 47 patients, 1 events; PSC- 69 patients, 12 events) reported incidence of IH at 18-months. Meta-analysis (Figure 7) demonstrated significant reduction of IH in the PMP group (OR=0.13 [0.02, 0.75]; p=0.02; I^2^=0%).

Incidence of IH at two years

IH incidence was reported by nine studies (PMP-936 patients, 114 events; PSC - 702 patients, 180 events) at the two-year follow-up. PMP significantly reduced the incidence in comparison to PSC (OR=0.23 [0.12, 0.45]; p<0.0001; I^2^=68%). Heterogeneity turned insignificant after removal of Sarr, 2014 study (new I^2^=47%; p=0.07). Leave one out analysis did not affect results.

No significant differences were noted in the following subgroups: (1) Study design (p-interaction=0.08; I^2^=67.2%), (2) Mesh location (p-interaction=0.77; I^2^=0%), (3) BMI (p-interaction=0.57; I^2^=0%), and (4) Study population (p-interaction=0.55; I^2^=0%).

**Figure 8:**
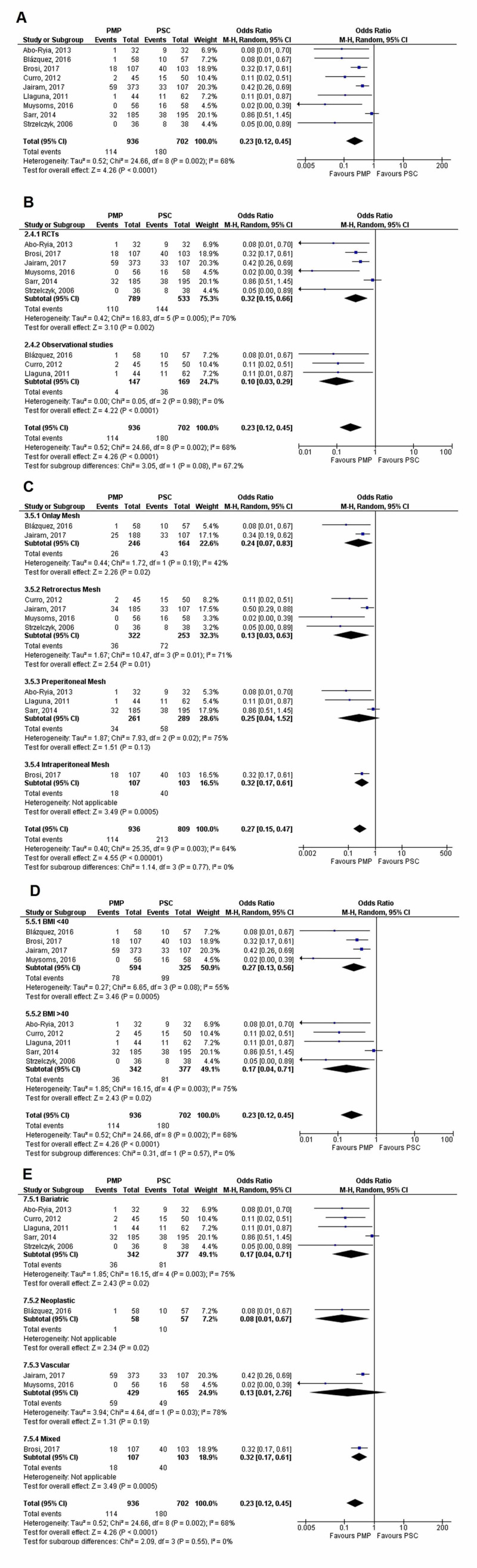
Incisional hernia at two years (A) Overall analysis; (B) Subgroups by study design; (C) Subgroups by mesh location; (D) Subgroups by BMI; and (E) Subgroups by population PMP, prophylactic mesh placement; PSC, primary suture closure; CI, confidence interval; M-H, Mantel-Haenszel Studies used in the analyses include [5,12,14,16,18,20-21,25,28].

Upon subgroup analysis by mesh location, however, all mesh locations except preperitoneal (OR=0.25 [0.04-1.52]; p=0.13; I^2^=75%) were found to reduce the risk of IH significantly. Subgroup analysis by study population showed that all populations except vascular (OR=0.13 [0.01, 2.76]; p=0.19; I^2^=78%) had a significant reduction in IH incidence after PMP. These findings are seen in Figure 8.

Incidence of IH at three years

Seven studies (PMP - 446 patients, 53 events; PSC - 466 patients, 122 events) reported the incidence of IH at three years. Mesh placement significantly decreased the IH incidence (OR=0.30 [0.16, 0.59]; p=0.0004; I^2^=52%). Sensitivity analysis by excluding individual studies kept results significant and robust. Heterogeneity (p=0.05) turned insignificant (p=0.80) and dropped to 0% after the removal of the Pans, 1998, study.

Upon subgroup analysis, no significant difference was found among: (1) Study design (p-interaction=0.21; I^2^=36.4%), and (2) BMI (p-interaction=0.15; I^2^=51.4%).

**Figure 9:**
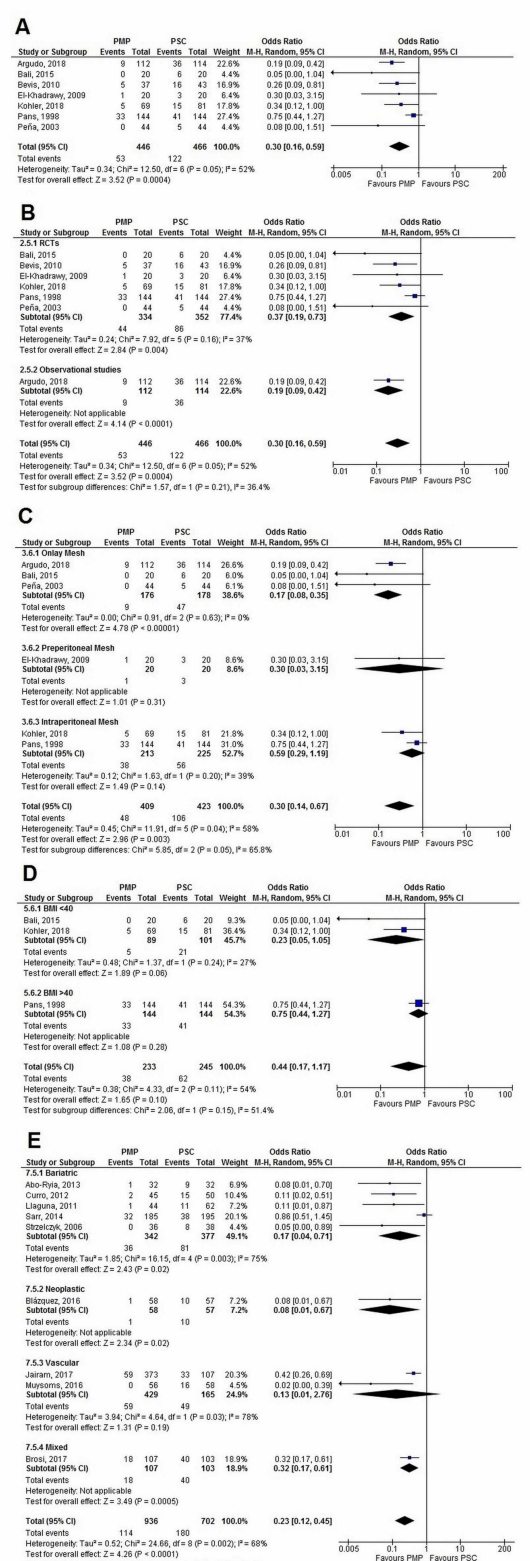
Incisional hernia at three years (A) Overall analysis; (B) Subgroups by study design; (C) Subgroups by mesh location; (D) Subgroups by BMI; and (E) Subgroups by population PMP, prophylactic mesh placement; PSC, primary suture closure; CI, confidence interval; M-H, Mantel-Haenszel Studies used in the analyses include [1,9,11,13,19,23-24].

On grouping data by mesh location and study population, a significant difference was seen between subgroups (p-interaction=0.05 and 0.02, respectively). While both PMP in the onlay position (OR=0.17 [0.08, 0.35]; p<0.00001; I^2^=0%) as well as the intraperitoneal position (OR=0.59 [0.29, 1.19]; p=0.14; I^2^=39%) reduced IH, performance in the onlay position was significantly better (p-interaction=0.02). All populations except bariatric (OR=0.71 [0.43, 1.20]; p=0.20; I^2^=0%) showed significant reduction in IH incidence at the three-years follow-up after PMP. Individual forest plots for all analyses are given in Figure 9.

The different subgroups analyses carried out for IH at the two-year and three-year follow-up are given in Table 5.

**Table 5:** Results of subgroup analyses for IH at the two-year and three-year follow-ups All outcomes are stratified according to study design (RCTs or observational), mesh location (onlay, retrorectus, preperitoneal, and intraperitoneal), mean BMI (<40 and >40), and study population (bariatric, neoplastic, vascular, and mixed). The value of I^2^ shows the heterogeneity among subgroups. P_subgroup_ represents p-values between subgroups. IH, incisional hernia; No Sig. Diff., no significant difference; Sig. Diff, Significant difference † - Significant difference was found only between onlay and intraperitoneal mesh (p=0.02). †† - Neoplastic group has a significantly lower incidence of IH than the bariatric group (p=0.006).

Subgroups	IH at 2-years follow-up					IH at 3-year follow-up				
	N studies	I^2^ (%)	OR [95% CI]	P_subgroup_	Comments	N studies	I^2^ (%)	OR [95% CI]	P_subgroup_	Comments
Study design										
RCT	6	70	0.32 [0.15, 0.66]	0.08	No Sig. Diff.	6	37	0.37 [0.19, 0.73]	0.21	No Sig. Diff.
Observational	3	0	0.10 [0.03, 0.29]			1	-	0.19 [0.09, 0.42]		
Mesh location										
Onlay	2	42	0.24 [0.07, 0.83]	0.77	No Sig. Diff.	3	0	0.17 [0.08, 0.35]	0.05†	Sig. Diff.
Retrorectus	4	71	0.13 [0.03, 0.63]			-	-	-		
Preperitoneal	3	75	0.25 [0.04, 1.52]			1	-	0.30 [0.03, 3.15]		
Intraperitoneal	1	-	0.32 [0.17, 0.61]			2	39	0.59 [0.29, 1.19]		
Mean BMI										
<40	4	55	0.27 [0.13, 0.56]	0.57	No Sig. Diff.	2	27	0.23 [0.05, 1.05]	0.15	No Sig. Diff.
>40	5	75	0.17 [0.04, 0.71]			1	-	0.75 [0.44, 1.27]		
Study population										
Bariatric	5	75	0.17 [0.04, 0.71]	0.55	No Sig. Diff.	2	0	0.71 [0.43, 1.20]	0.02††	Sig. Diff.
Neoplastic	1	-	0.08 [0.01, 0.67]			1	-	0.19 [0.09, 0.42]		
Vascular	2	78	0.13 [0.01, 2.76]			2	0	0.22 [0.08, 0.62]		
Mixed	1	-	0.32 [0.17, 0.61]			2	0	0.29 [0.11, 0.79]		

Incidence of IH at five years

**Figure 10:**
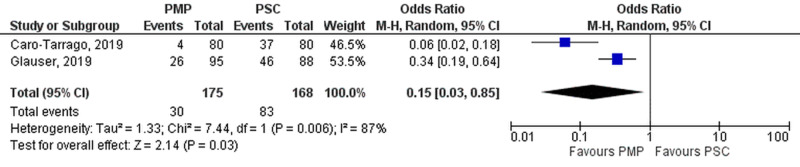
Incisional hernia at five years PMP, prophylactic mesh placement; PSC, primary suture closure; CI, confidence interval; M-H, Mantel-Haenszel Studies used in the analysis include [2-3].

Follow-up at five years was reported in two studies (PMP - 175 patients, 30 events; PSC - 168 patients, 83 events), and pooled results significantly favored PMP (OR=0.15 [0.03, 0.85]; p=0.03; I^2^=87%) as seen in Figure 10.

Seroma

A total of 19 studies (PMP - 1290 patients, 127 events; PSC - 1292 patients, 70 events) reported postoperative seroma details. We accepted the investigators’ definition of seroma. PSC significantly reduced the incidence of seroma when compared to PMP (OR=1.67 [1.10, 2.55]; p=0.02; I^2^=19%). Individual removal of either the Caro-Tarrago, 2019, or Jairam, 2017 study on the leave one out analysis turned results insignificant.

Upon subgroup analysis, a significant difference was noted between study population subgroups (p-interaction=0.04; I^2^=64.5%). PSC had a significantly reduced incidence of seroma in all study populations except the mixed subgroup (OR=0.47 [0.17, 1.27]; p=0.14; I^2^=0%).

**Figure 11:**
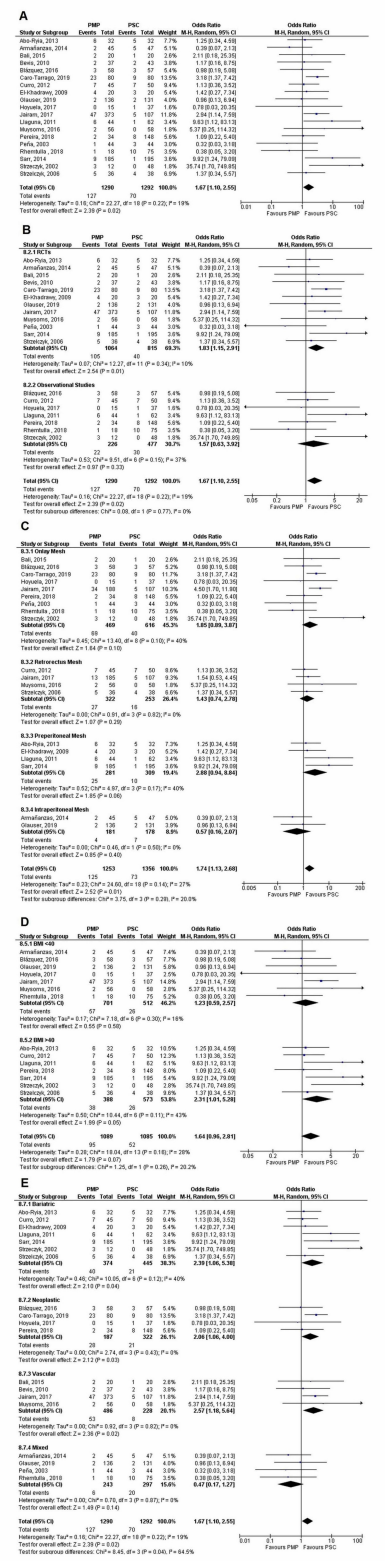
Seroma (A) Overall analysis; (B) Subgroups by study design; (C) Subgroups by mesh location; (D) Subgroups by BMI; and (E) Subgroups by population PMP, prophylactic mesh placement; PSC, primary suture closure; CI, confidence interval; M-H, Mantel-Haenszel Studies used the analyses include [1-3,5-6,10-22,25].

However, no statistically significant difference was found upon classifying data into: (1) Study design (p-interaction=0.77; I^2^=0%), (2) Mesh location (p-interaction=0.29; I^2^=20 %), and (3) BMI (p-interaction=0.26; I^2^=20.2 %). Forest plots reporting these findings are displayed in Figure 11.

Chronic wound pain

Six studies (PMP - 346 patients, 45 events; PSC - 395 patients, 29 events) reported the incidence of chronic wound pain. The PSC group had significantly reduced chronic wound pain as compared to PMP (OR=1.71 [1.03, 2.83]; p=0.04; I^2^=0%). Individual removal of (a) El-Khadrawy, 2009, (b) Muysoms, 2016, (c) Sarr, 2014, and (d) Strzeczyk, 2002, made results insignificant.

**Figure 12:**
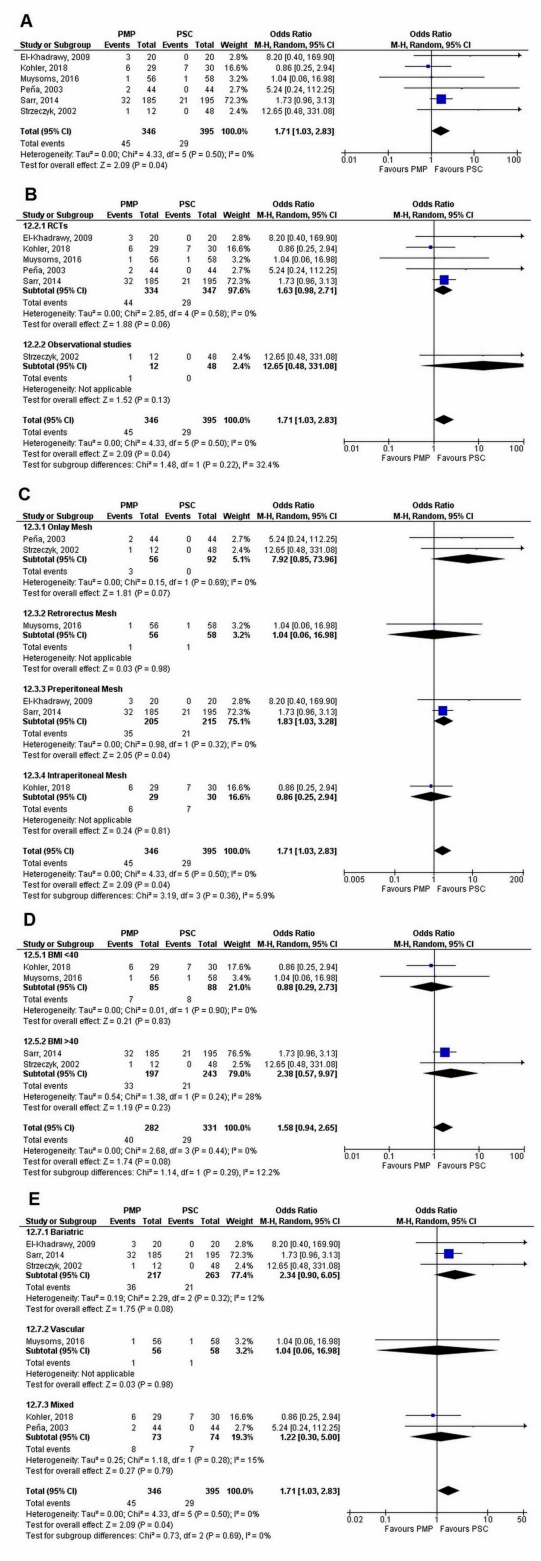
Chronic wound pain (A) Overall analysis; (B) Subgroups by study design; (C) Subgroups by mesh location; (D) Subgroups by BMI; and (E) Subgroups by population PMP, prophylactic mesh placement; PSC, primary suture closure; CI, confidence interval; M-H, Mantel-Haenszel Studies used in analyses include [1,10-11,18,20,23].

Our results were robust and no significant difference among subgroups was found when data was stratified into: (1) Study design (p-interaction=0.22; I^2^=32.4%), (2) Mesh location (p-interaction=0.36; I^2^=5.9%), (3) BMI (p-interaction=0.29; I^2^=12.2%), and (4) Study population (p-interaction=0.69; I^2^=0%). Figure 12 displays the forest plots of all subgroup analyses for chronic wound pain.

Table 6 reports the subgroup analysis carried out for seroma and chronic wound pain.

**Table 6:** Results of subgroup analyses for seroma and chronic wound pain All outcomes are stratified according to study design (RCTs or observational), mesh location (onlay, retrorectus, preperitoneal, and intraperitoneal), mean BMI (<40 and >40), and study population (bariatric, neoplastic, vascular, and mixed). The value of I^2^ shows the heterogeneity among subgroups. P_subgroup_ represents p-values between subgroups. IH, incisional hernia; OR, odds ratio; No Sig. Diff., no significant difference; Sig. Diff, significant difference †- Significant difference was found between the mixed group with the bariatric (p=0.01), neoplastic (p=0.02), and vascular (p=0.008) groups.

Subgroups	Seroma					Chronic wound pain				
	N studies	I^2^ (%)	OR [95% CI]	P_subgroup_	Comments	N studies	I^2^ (%)	OR [95% CI]	P_subgroup_	Comments
Study design										
RCT	12	10	1.83 [1.15, 2.91]	0.77	No Sig. Diff.	5	0	1.63 [0.98, 2.71]	0.22	No Sig. Diff.
Observational	7	37	1.57 [0.63, 3.92]			1	-	12.65 [0.48, 331.08]		
Mesh location										
Onlay	9	40	1.85 [0.89, 3.87]	0.29	No Sig. Diff.	2	0	7.92 [0.85, 73.96]	0.36	No Sig. Diff.
Retrorectus	4	0	1.43 [0.74, 2.78]			1	-	1.04 [0.06, 16.98]		
Preperitoneal	4	40	2.88 [0.94, 8.84]			2	0	1.83 [1.03, 3.28]		
Intraperitoneal	2	0	0.57 [0.16, 2.07]			1	-	0.86 [0.25, 2.94]		
Mean BMI										
<40	7	16	1.23 [0.59, 2.57]	0.26	No Sig. Diff.	2	0	0.88 [0.29, 2.73]	0.29	No Sig. Diff.
>40	7	43	2.31 [1.01, 5.28]			2	28	2.38 [0.57, 9.97]		
Study population										
Bariatric	7	40	2.39 [1.06, 5.38]	0.04†	Sig. Diff.	3	12	2.34 [0.90, 6.05]	0.69	No Sig. Diff.
Neoplastic	4	0	2.06 [1.06, 4.00]			0	-	-		
Vascular	4	0	2.57 [1.18, 5.64]			1	-	1.04 [0.06, 16.98]		
Mixed	4	0	0.47 [0.17, 1.27]			2	15	1.22 [0.30, 5.00]		

Sensitivity analysis by excluding non-midline incisions and laparoscopic surgeries

**Figure 13:**
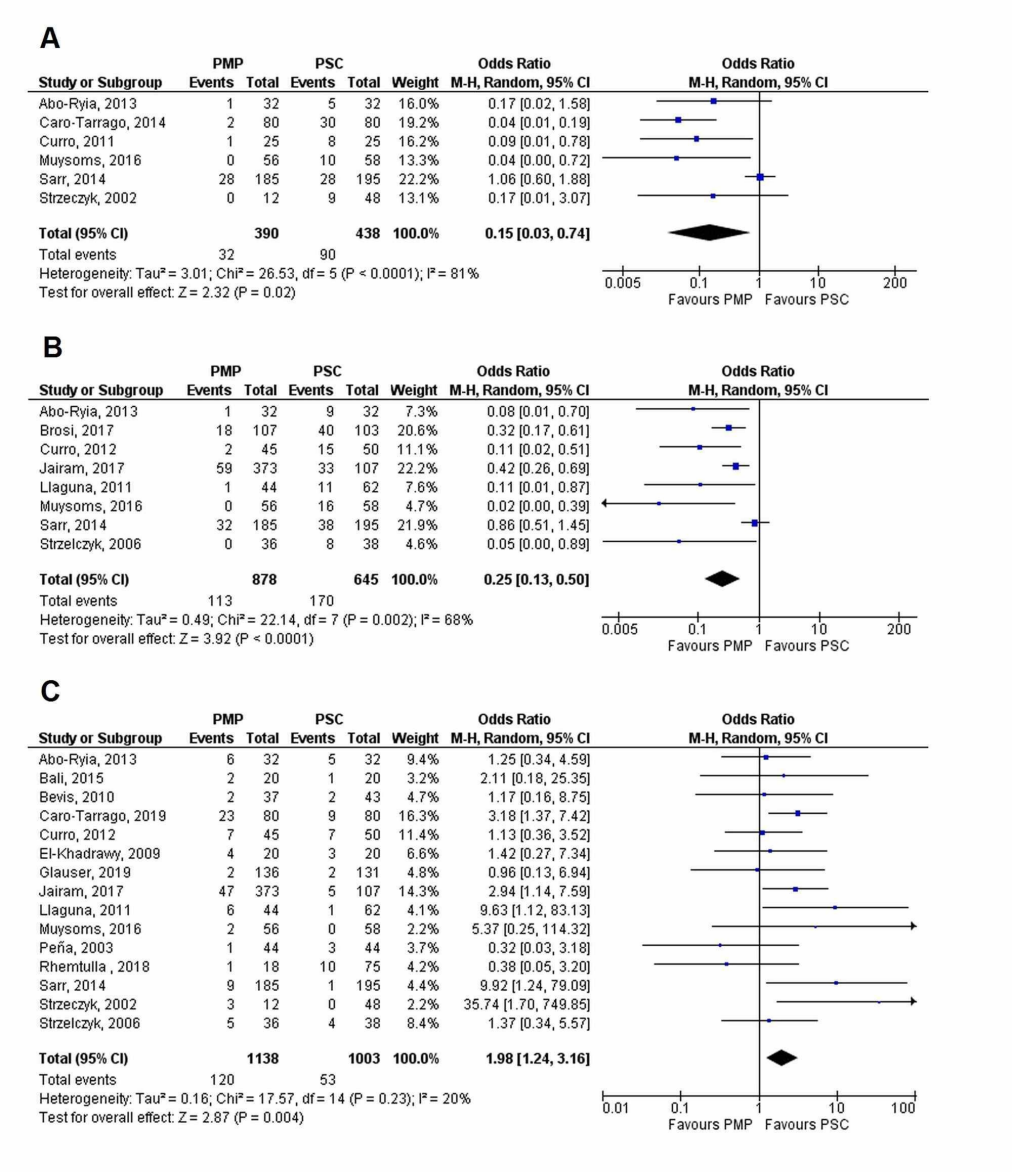
Forest plot showing the results of the sensitivity analysis by excluding non-midline incisions and laparoscopic surgeries (A) IH at 1-year; (B) IH at 2-year; and (C) Seroma IH, incisional hernia; PMP, prophylactic mesh placement; PSC, primary suture closure; CI, confidence interval; M-H, Mantel-Haenszel

Additional sensitivity analyses were done by excluding studies that employed non-midline incisions or laparoscopic procedures for outcomes, namely risk of IH at one year and two years, and seroma (Figure 13). The three-year and five-year follow-up data for IH and chronic wound pain did not include non-midline incision studies, so they were exempted from this sensitivity analysis. Results did not differ significantly after sensitivity analysis and PMP was still found to significantly reduce the risk of IH at the one-year (OR=0.15 [0.03, 0.74]; p=0.02; I^2^=81%) and two-year (OR=0.25 [0.13, 0.50]; p<0.0001; I^2^=68%) follow-ups, but it significantly increased the risk of seroma (OR=1.98 [1.24, 3.16]; p=0.004, I^2^=20%).

Other secondary outcomes

**Figure 14:**
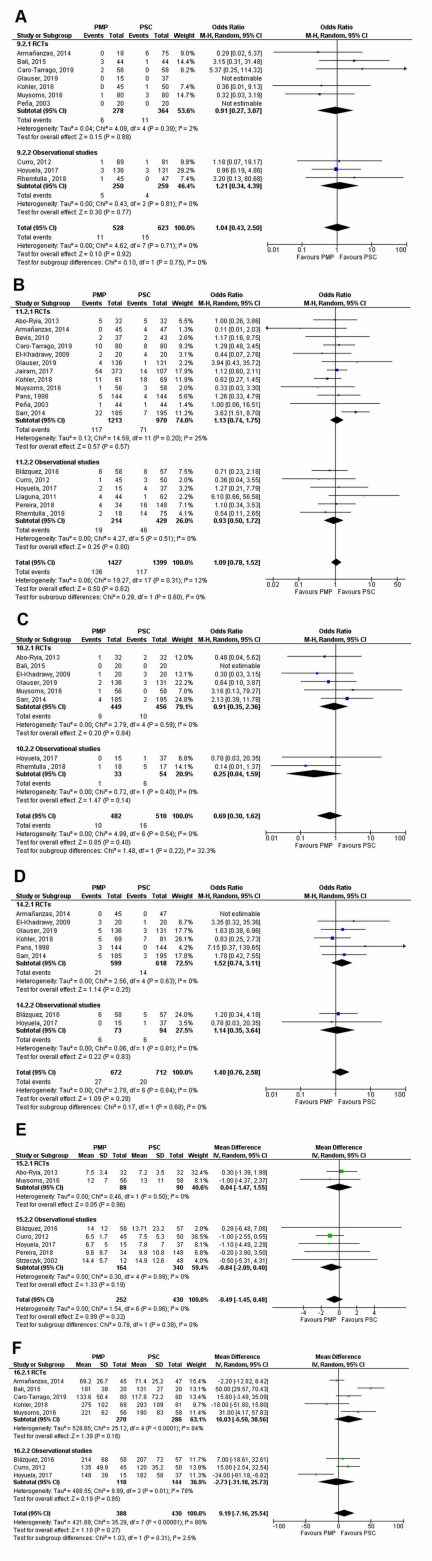
Forest plot showing results of other secondary outcomes (A) Hematoma; (B) Surgical site infection; (C) Wound dehiscence; (D) Gastrointestinal complications; (E) Length of hospital stay; and (F) Operating time PMP, prophylactic mesh placement; PSC, primary suture closure; CI, confidence interval; M-H, Mantel-Haenszel Studies used in the analyses include [1-3,5,9-29].

No significant difference was seen between the PMP and PSC groups in risk of postoperative hematoma (OR=1.04 [0.43, 2.50]; p=0.92; I^2^=0%), surgical site infection (OR=1.09 [0.78, 1.52]; p=0.62; I^2^=12%), wound dehiscence (OR=0.69 [0.30, 1.62]; p=0.40; I^2^=0%), gastrointestinal complications (OR=1.40 [0.76, 2.58]; p=0.28; I^2^=0%), length of hospital stay (WMD=-0.49 [-1.45, 0.47]; p=0.32; I^2^=0%), and operating time (WMD=9.18 [-7.17, 25.53]; p=0.27; I^2^=80%). The individual forest plots for all above-mentioned outcomes are given in Figure 14. There was no subgroup difference when all secondary outcomes were stratified according to study design as shown in Table 7

**Table 7:** Results of subgroup analyses for secondary outcomes P_subgroup_ represents p-values between subgroups. IH, incisional hernia; I^2^, heterogeneity; OR, odds ratio; CI, confidence interval; No Sig. Diff., no significant difference

Outcomes	RCT			Observational studies			P_subgroups_	Comments
	N studies	I^2^ (%)	OR [95% CI]	N studies	I^2^ (%)	OR [95% CI]		
Hematoma	7	2	0.91 [0.27, 3.07]	3	0	1.21 [0.34, 4.39]	0.75	No Sig. Diff.
Surgical Site Infection	12	25	1.13 [0.74, 1.75]	6	0	0.93 [0.50, 1.72]	0.60	No Sig. Diff.
Wound dehiscence	6	0	0.91 [0.35, 2.36]	2	0	0.25 [0.04, 1.59]	0.22	No Sig. Diff.
Gastrointestinal complications	6	0	1.52 [0.74, 3.11]	2	0	1.14 [0.35, 3.64]	0.68	No Sig. Diff.
Operating time (minutes)	5	84	16.03 [-6.51, 38.56]	3	78	-2.73 [-31.18, 25.73]	0.31	No Sig. Diff.
Length of Hospital stay (days)	2	0	0.04 [-1.47, 1.55]	5	0	-0.84 [-2.09, 0.40]	0.38	No Sig. Diff.

## Discussion

Our study shows that PMP offers a greater benefit in the prevention of IH than PSC. Mesh placement showed a reduction in IH risk at intervals of one-year, 18-months, two-years, three-years, and five-years postoperatively. On subgroup analysis, only study population and mesh location were found to influence hernia development.

PMP reduced the risk of IH in most populations, with a few exceptions. At an interval of one- and three-years, the mesh failed at IH prevention in the bariatric group while in the vascular subgroup, the mesh resulted in being inefficacious at the two-year interval. This was in contrast to the meta-analyses by Dasari M et al. (bariatric) and Timmermans et al. (abdominal aortic aneurysm) [7,30]. The exact reason for the difference in findings is unclear, but it was noted that most of the included studies in the previous meta-analyses had a shorter time interval of six months to one year as compared to ours, which evaluated for a longer follow-up of two to three years [7,30]. For shorter-term periods of six months, PMP was inefficacious at IH prevention. Only two studies have inspected the outcomes at a five-year interval [2-3]. However, to determine the accurate recurrence following IH repair, we recommend that patients should be followed for a longer period of time (10-15-year follow-up). 

Not all mesh locations were effective at IH prevention, and the best effect was observed with onlay and intraperitoneal mesh placement. The onlay position was superior to intraperitoneal in efficacy but with higher complication rates. Midline and lateral incisions are best reinforced when onlay mesh placement is adopted. Though not extensively discussed, some studies hinted at the superior efficacy of the onlay position [4,8]. The safety and efficacy of mesh type were not extensively studied in our meta-analysis, as they were beyond the scope of our discussion.

Among the secondary outcomes, only the risk of seroma and chronic wound pain were found to be significantly increased in the PMP group. Seroma was significantly increased (about two times) in those with PMP. This concurred with the analysis by Borab et al. and Wang et al. but was contrary to Timmermans et al., where no such difference was observed [4,7-8]. Upon further subgroup analysis, only onlay positioning had approximately thrice the risk of seroma development. Borab M et al. reported that onlay and preperitoneal PMP were linked with a higher risk of seroma development, which was further aggravated when the PP mesh was placed in the onlay position [4]. This may be well-explained by the extensive dissection in onlay position, thus increasing the likelihood of postoperative complications.

Most seroma cases were less morbid and were treated conservatively with antibiotics and percutaneous drainage. However, some mentioned the removal of mesh due to infection [13,28]. To decrease seroma incidence, subcutaneous drainage and appropriate tissue management were advised [6].

Chronic wound pain significantly impacts QoL in patients after any surgical procedure. The degree of pain is closely associated with the type and extent of surgery, nerve damage, intensity of radio and chemotherapy, and psychosocial factors. The risk of chronic wound pain with mesh placement is of much conjecture, as few studies reported a lower incidence of chronic wound pain with mesh use, but the meta-analysis by Wang et al. found that mesh failed to provide any significant reduction in chronic wound pain [8]. The results of Wang et al. were limited by a small sample size (3 studies; 229 participants) [8]. Our analysis (6 studies; 741 participants) showed that mesh was associated with a significantly increased incidence of chronic wound pain compared to suture closure. To quantify the debilitating burden of chronic pain, few studies included standardized scoring such as visual analog scale (VAS) for pain and the EQ-5D (EuroQol- 5 Dimension) and SF-36 (36-Item Short Form Survey) questionnaires for QoL [5,20]. No difference in QoL was found between the mesh and suture groups [5,20]. Patients in both the PSC and PMP groups suffered from chronic pain, which, however, was well-tolerated and rarely interfered with routine activities, hence resulting in higher patient satisfaction [20].

The difference in other secondary outcomes was non-significant. SSI, an infrequent complication in mesh hernioplasties, is influenced by certain risk factors such as mesh type, obesity, smoking history, mean operative time, and degree of emergency [5,16,20,25]. The lack of a significant difference between PMP and PSC for SSI could be attributed to a few postulates. Firstly, only a few studies adopted complication assessment protocols and standardized assessment scales to gauge the effect of PMP on different outcomes and QoL. Secondly, there may be an underestimation of the additional complications due to underreporting.

No significant difference was observed for the length of hospital stay and mean operative time in our study, whereas Wang et al. found mesh use to be associated with increased operative time [8]. Though insignificant, the results showed a trend of reduced length of hospital stay with mesh use but with increased operative time. Additionally, studies failed to analyze the effect of strenuous activities and early resumption of work. We excluded ‘reoperation’ and 're-hospitalization' outcomes due to discrepancies in the defining criteria. 

Strengths, limitations, and future suggestions

To the best of our knowledge, this is the first study to run a follow-up duration-based analysis of IH and included clinical outcomes in various patient populations.

This updated analysis has adopted a more integrated, extensive (including both observational studies and RCTs), and comparative approach to gain better insight into the outcomes. Subgroup analysis for IH outcomes at different follow-up intervals and other significant outcomes (seroma and chronic wound pain) may help predict the postoperative outcomes better.

However, some aspects may have been missed owing to insufficient studies, ambiguous reporting, or the redundancy of the results. There is a lack of universal agreement on the definition of hernia recurrence and the indications for surgical repair. To establish the long-term viability of mesh, the effect of materials and techniques on the outcomes needs to be addressed. Furthermore, the efficacy of mesh type and its location needs to be extensively evaluated.

Since this only catered to elective cases, a comparison analysis between emergency and elective procedures and among other hernia types is warranted. Additionally, the influence of the surgeon’s technique and expertise on postoperative outcomes is less frequently addressed and studies should be carried out to explore this aspect as well. These measures may assist in bridging the major gaps in clinical practice.

Clinical implications

Mesh placement has proven to be, repeatedly, effective in decreasing the incidence of IH after elective midline laparotomy and laparoscopy. This stands true for some cases of parastomal hernia and emergency laparotomy. Even with existing infection, mesh use is associated with better results in both hernia prevention and in lowering wound morbidity [2,10].

Additionally, the adoption of a benefit vs. risk approach in vulnerable (high-risk) patient populations may assist in reducing the complications. In one of the included studies by N Argudo et al. (2018), the selection of patients for mesh placement utilizing an algorithm decreased the recurrence of the hernia, lowered the number of complications, and saved a considerable cost burden [24]. Therefore, a standardized approach for mesh placement can assist in lowering the cost burden and in decreasing the mortality rates.

## Conclusions

PMP has been effective in decreasing the recurrence rates of IH for both shorter and longer time periods. It is, however, associated with an increased incidence of seroma and chronic wound pain. No significant difference was found between the PMP and PSC groups for hematoma, surgical site infection, wound dehiscence, gastrointestinal complications, length of hospital stay, and operating time. The benefits of PMP largely outweigh the risk of complications and is beneficial for high-risk patient populations. There is a need for trials with extensive follow-up durations of 10-15 years to study the long-term benefits of mesh, and more studies with uniform reporting criteria are needed for accurately analyzing chronic wound pain outcomes. Furthermore, studies evaluating the efficacy of one mesh type over another are warranted.

## Appendices

**Table 8:** Incisional hernia diagnosis and details of mesh, suture, and surgery for included studies IH, incisional hernia; CT, computed tomography

Study, year	Diagnosis of IH	Mesh location	Mesh material	Suture for closing aponeurosis	Technique for closing aponeurosis
Pans, 1998 [9]	Physical exam	Intraperitoneal	Polyglactin	Polyglactin	-
Strzeczyk, 2002 [10]	-	Onlay	Polypropylene	Polypropylene 1	Continuous
Peña, 2003 [11]	Physical exam & CT scan	Onlay	Polypropylene	Nonabsorbable filament	Continuous
Strzelczyk, 2006 [12]	Ultrasound	Retrorectus	Polypropylene	Polypropylene 2	Continuous
El- Khadrawy, 2009 [1]	Ultrasound	Preperitoneal	Polypropylene	Polypropylene 1	Continuous
Bevis, 2010 [13]	Clinical exam or ultrasound	Preperitoneal & retrorectus	Polypropylene	Nonabsorbable filament	-
Llaguna, 2011 [14]	Physical exam & imaging studies	Preperitoneal	Biologic (Alloderm)	Polydioxanone 1	Continuous
Curro, 2012 [15]	Clinical exam or ultrasound	Retrorectus	Polypropylene	Polyglactin & polydiossanone	Interrupted
Abo-Ryia, 2013 [16]	Clinical exam or ultrasound	preperitoneal	Polypropylene	Polypropylene 1	Continuous
Armañanzas, 2014 [17]	Clinical exam or CT scan	intraperitoneal	Polypropylene	Nonabsorbable polyester	-
Sarr, 2014 [18]	Clinical exam & imaging modality.	Preperitoneal	Biologic (Surgisis Gold)	Nylon, polypropylene, & polydioxanone	Continuous
Bali, 2015 [19]	Clinical exam or CT scan	Onlay	Biologic (bovine pericardium)	Polydioxanone 1 loop	Continuous
Muysoms, 2016 [20]	Clinical exam, CT scan, or Ultrasound	Retrorectus	Polypropylene	Polydioxanone	-
Blázquez, 2016 [21]	CT scan	Onlay	Propylene polyglycolic acid	Poly 4 hydroxybutyrate	2 layer closure
Jairam, 2017 [5]	Physical exam, ultrasound, or CT scan	Onlay & Retrorectus	Polypropylene (Optilene)	Slow absorbable with loop	Continuous
Hoyuela, 2017 [22]	Clinical exam or CT scan	onlay	Polypropylene	Absorbable monofilament	Continuous
Kohler, 2018 [23]	Clinical exam or imaging studies	intraperitoneal	Polypropylene-polyvinylidene fluoride	Slow absorbable	Continuous
Argudo, 2018 [24]	Clinical diagnosis or CT scan	Onlay	Low weight, wide pore, partially absorbable	Slowly absorbable	Continuous
Pereira, 2018 [25]	Clinical exam or CT scan	Onlay	Polyvinylidenefluoride mesh	Polydioxanone gauge loop	Continuous
Rhemtulla, 2018 [6]	CT scan	Onlay	Biosynthetic mesh	Heavy, slow absorbing	Continuous, accompanied by short stitch technique with 5-7mm bites
Glauser, 2019 [2]	Clinical exam or ultrasound	Intraperitoneal	Absorbable Porcine collagen, polyethylene glycol, glycerol	Late absorbable monofilament polydioxanone loop suture	Continuous
Caro‑Tarrago, 2019 [3]	Clinical exam or CT scan	Onlay	Polydioxanone loop, propylene mesh	Polydioxanone 1	Continuous

**Table 9:** Cochrane tool for assessing risk of bias in RCTs RCTs, randomized controlled trials Quality assessment of published studies included in the meta-analysis.

Study, year	Sequence generation	Allocation concealment	Blinding of participants	Blinding of outcome assessment	Incomplete outcome data	Selective outcome reporting	Other sources of bias
Pans, 1998 [9]	Unclear	Unclear	Unclear	Low	Low	Low	Low
Peña, 2003 [11]	Unclear	Unclear	Unclear	Unclear	Low	Low	Low
Strzelczyk, 2006 [12]	Low	High	High	Low	Low	Low	Low
El- Khadrawy, 2009 [1]	Unclear	Unclear	Unclear	Unclear	Low	Low	Low
Bevis, 2010 [13]	Low	Low	High	High	Low	Low	Low
Abo-Ryia, 2013 [16]	Unclear	Unclear	Low	Low	Low	Low	low
Armañanzas, 2014 [17]	Low	Low	Low	Low	Low	Low	Low
Sarr, 2014 [18]	Low	Low	Unclear	Unclear	Low	Low	low
Bali, 2015 [19]	Low	Unclear	Unclear	Unclear	Low	Low	Low
Muysoms, 2016 [20]	Low	Low	Low	High	Low	Low	Low
Jairam, 2017 [5]	Low	Low	Low	Low	Low	Low	Low
Kohler, 2018 [23]	Low	Low	Low	Low	Low	Low	Low
Glauser, 2019 [2]	Low	Low	Low	Low	Low	Low	Low
Caro‑Tarrago, 2019 [3]	Low	Low	Low	Low	Low	Low	Low

**Table 10:** Leave one out sensitivity analysis results for all outcomes

Outcome	Leave one out analysis results
IH at 6-months	No significant effect
IH at 1-year	No significant effect
IH at 18-months	Not applicable
IH at 2-year	No significant effect
IH at 3-years	Sensitivity analysis by excluding individual studies kept results significant and robust. Heterogeneity (p=0.05) turned in-significant (p=0.80) but dropped to 0% after removal of Pans, 1998 study.
IH at 5-year	Not applicable
Seroma	Removal of either Caro-Tarrago, 2019 or Jairam, 2017 study turned results insignificant {(OR=1.52 [0.97, 2.37]; p=0.07; I^2^= 15%) and (OR=1.55 [0.99, 2.44]; p=0.06; I^2^= 19%), respectively}.
Hematoma	No significant effect
Surgical site infection	No significant effect
Chronic wound pain	Results turned insignificant on individual removal of
	(a) El-Khadrawy, 2009, (New OR= 1.63 [0.98, 2.72]; p= 0.06; I^2^=0%)
	(b) Muysoms, 2016, (New OR=1.76 [0.99, 3.13]; p=0.06; I^2^=5%)
	(c) Sarr, 2014, and (New OR= 1.83 [0.63, 5.27]; p=0.26; I^2^= 8%)
	(d) Strzeczyk, 2002 (New OR=1.63 [0.98, 2.71]; p=0.06; I^2^= 0%)
Wound dehiscence	No significant effect
Gastrointestinal complications	No significant effect
Operating time	No significant effect
Hospital stay length	No significant effect
